# Supplementary material for: Reovirus Type 3 Dearing Variants Do Not Induce Necroptosis in RIPK3-Expressing Human Tumor Cell Lines
Source: Int J Mol Sci. 2023 Jan 24;24(3):2320. doi: 10.3390/ijms24032320 (PMC9916669; doi:10.3390/ijms24032320)
Supplement: Supplementary file 1 [file ijms-24-02320-s001.zip › Figures.pdf]

Reovirus Type 3 Dearing variants do not induce necroptosis in RIPK3-expressing human tumor cell lines

Supplemental data

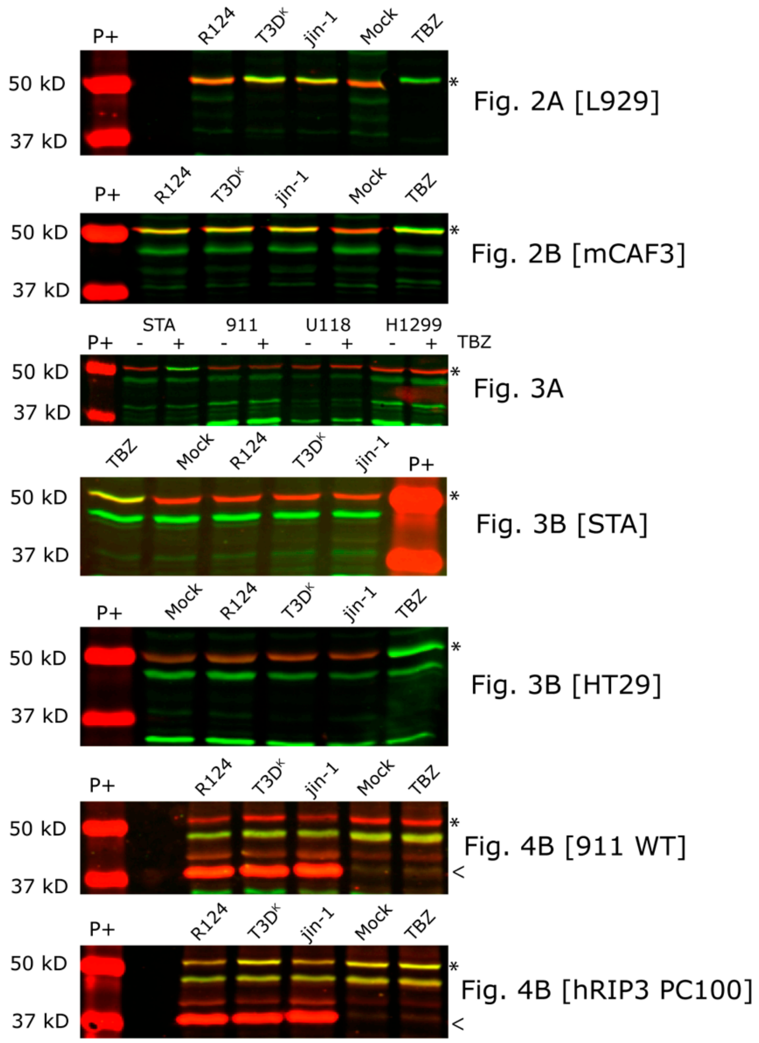

Figure S1. Color panels of all blots as shown in the manuscript. Blots of corresponding figures were stained for phosphorylated MLKL using primary antibodies RαM-pMLKL (mouse phospho S345, and human phospho S358) and detected with secondary IRDye DoαR 800CW (green). MLKL total protein was assessed with Rat anti-MLKL, clone 3H1 (both species) and detected with IRDye GαRat 680RD (red). For the panel in figure 4, σ3 was detected with antibody 4F2 and secondary antibody DoαM 680 RD (red). Bands in the P+ lane are from the All Blue Precision Plus marker (Bio-Rad). STA is STA-et2.1, TBZ is a cocktail of TNFα, BV6 and Z-VAD. Asterisks (*) indicate p-MLKL/MLKLtot signal, and < in the panel of figure 6A corresponds with σ3 signal.

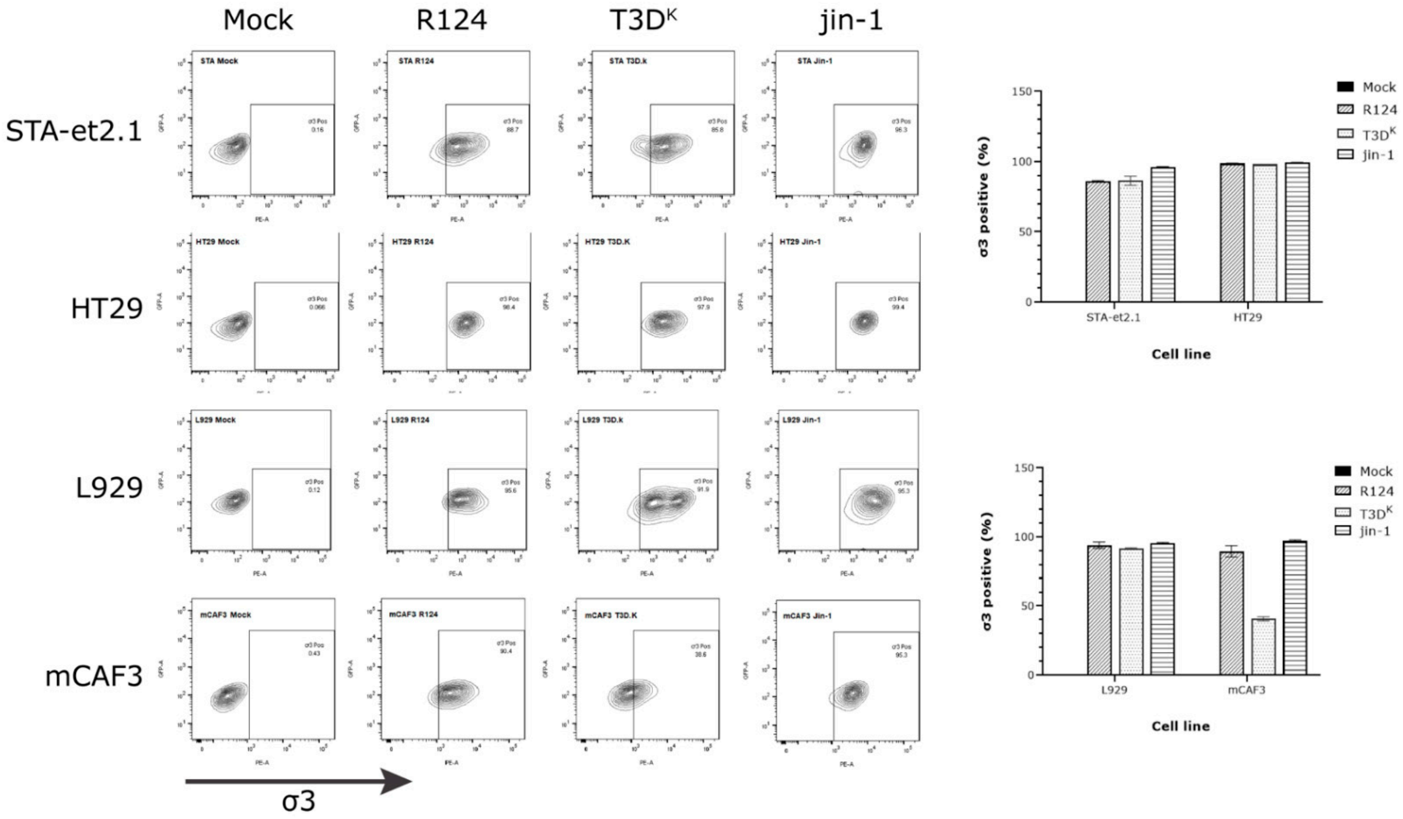

Figure S2. Detection of σ3-positive cells by flow cytometry. Cells in 24-well plates were exposed to wt-R124, T3D^K and jin-1 viruses (MOI = 10 for L929 and mCAF-3 cells or MOI = 50 for STA-et2.1 and HT29 cells) or mock in DMEM-2% FBS, in duplicate. Cells were fixed 30 hr post-infection with fixative from BD Cytofix/Cytoperm kit (BD Biosciences, Vianen, The Netherlands). After one wash with the accompanied BD Permeabilization/Wash buffer (PW buffer), cells were permeabilized in PW buffer. Cells were stained with σ3 antibody (4F2), 1:500, diluted in PW buffer followed by secondary PE-conjugated, anti-Mouse IgG antibody (12-4010-87, 1:1000 USB Molecular Biology Reagents: VWR International, Amsterdam, The Netherlands) in PW buffer. Before analysis with an LSR II flow cytometer (BD Biosciences), cells were taken up in FACS buffer (PBS with 0.5% BSA and 2 mM EDTA). The percentage of σ3-positive cells was determined using FlowJo software (V10; Treestar). Plots are an example of the gating of one series. Graphs represent one experiment with two independent infections, error bars are standard deviations (SD).

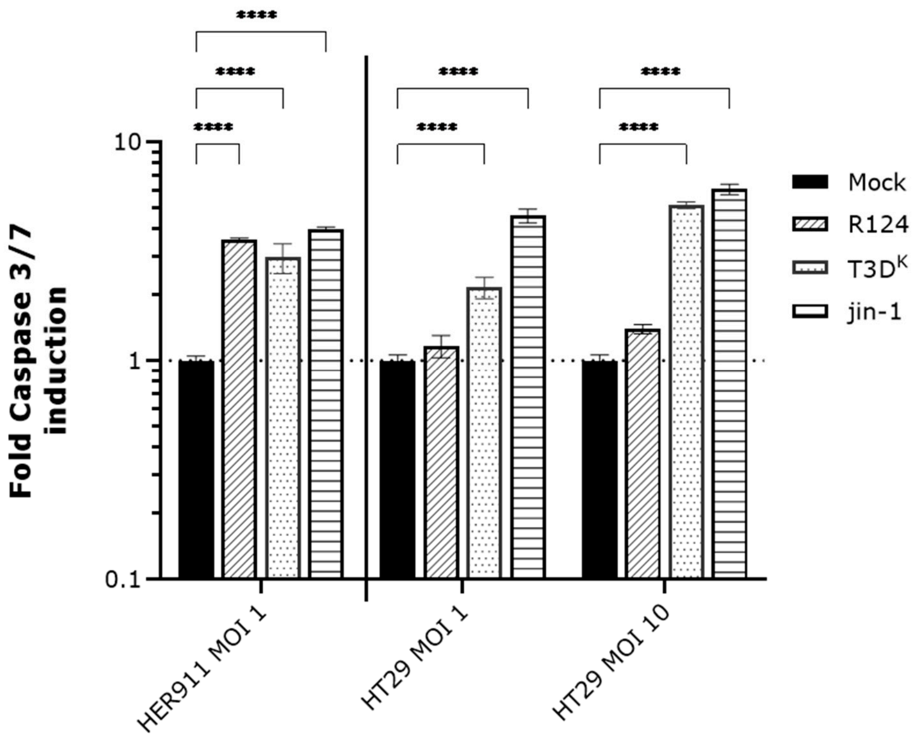

Figure S3. Induction of caspase 3/7 upon reovirus infection in HT29 cells. Cells in 96-well format were mock-infected or infected with wt-R124, T3D^K and jin-1 with indicated MOIs (triplicates). HER911 cells were taken along as a positive control and with MOI = 1 only. Caspase 3/7 activity was measured 40 hr post-infection with the Caspase-Glo 3/7 detection kit according to the accompanied manual (Promega, Leiden, The Netherlands). The fold induction was normalized to mock-infected cells. Statistical significance is determined by two-way ANOVA with Bonferroni's multiple comparisons: **** $p<0.0001$.

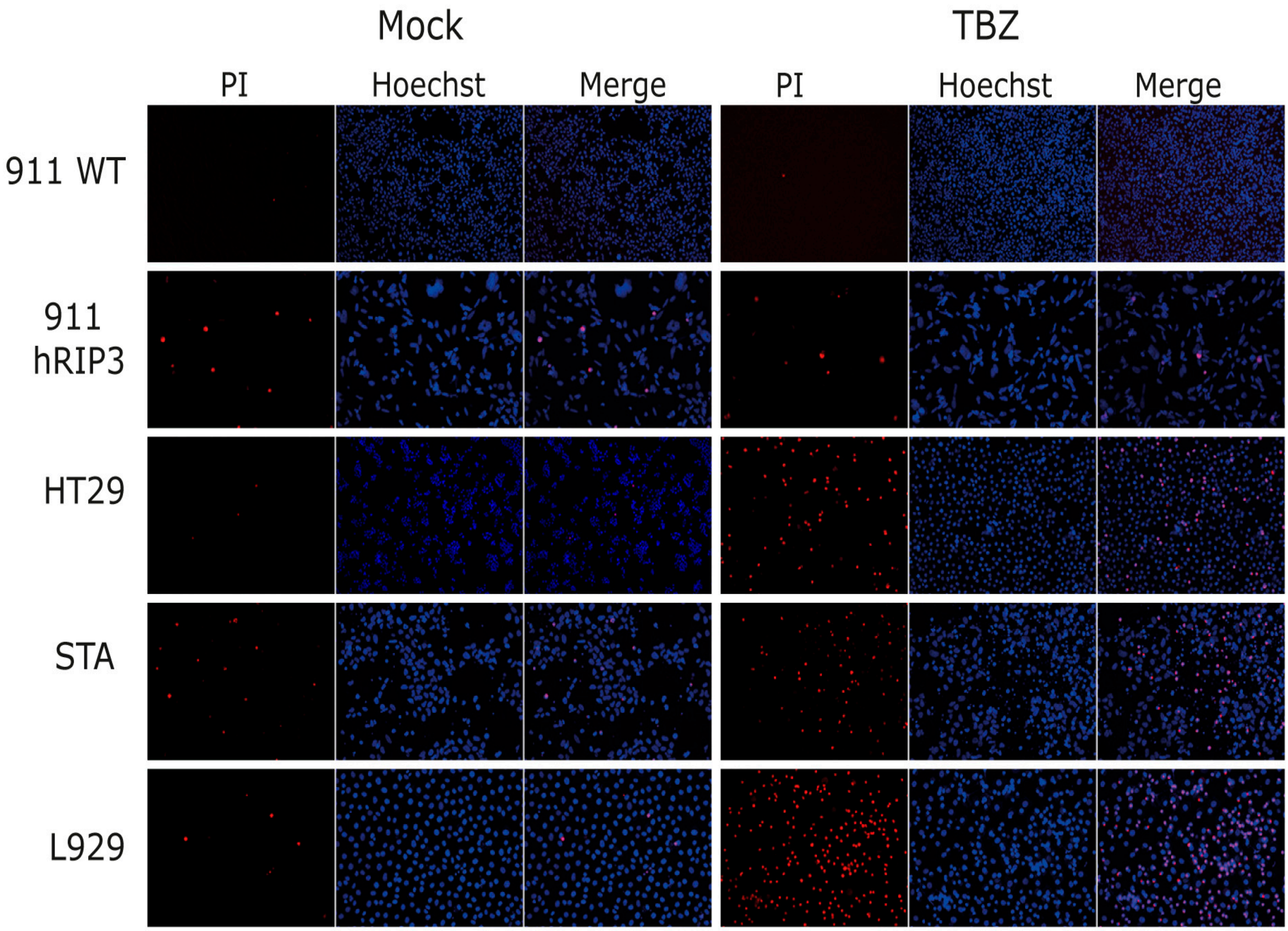

Figure S4. Propidium Iodide (PI) uptake in mock-treated vs TBZ-treated cells. Representative example of PI/Hoechst staining, used as input for calculations of PI-positive cells (Figure 4C). HER911 cells (911 WT) and HER911hRIP3 PC100 (911 hRIP3), HT29 and STA-et2.1 cells were treated or not with TBZ for 24 hours before staining with PI and Hoechst 33342. Immediately after staining, pictures of PI and Hoechst signal were taken. The pictures served as input for counting PI- and Hoechst-positive cells with Fiji scripts.

Fiji scripts

Script used for PI staining:

```
makeRectangle(0, 0, 2560, 1826);
run("Crop");
run("8-bit");
setOption("BlackBackground", false);
setThreshold(33, 255);
run("Convert to Mask");
run("Watershed");
run("Analyze Particles...", "size=10-Infinity circularity=0.50-1.00 show=Outlines exclude
clear summarize");
selectWindow("Threshold");
run("Close");
```

Script used for Hoechst staining:

```
makeRectangle(0, 0, 2560, 1820);
run("Crop");
run("8-bit");
setOption("BlackBackground", false);
setThreshold(40, 255);
run("Convert to Mask");
run("Watershed");
run("Analyze Particles...", "size=10-Infinity circularity=0.10-1.00 show=Outlines exclude
clear summarize");
selectWindow("Threshold");
run("Close");
```

Figure S5. Fiji scripts for calculation PI-positive cells.
